# Analysis of the impact of handling and culture on the expansion and functionality of NK cells

**DOI:** 10.3389/fimmu.2023.1225549

**Published:** 2023-08-11

**Authors:** Sara Martin-Iglesias, Lara Herrera, Silvia Santos, Miguel Ángel Vesga, Cristina Eguizabal, Senentxu Lanceros-Mendez, Unai Silvan

**Affiliations:** ^1^ BCMaterials, Basque Center for Materials, Applications and Nanostructures, UPV/EHU Science Park, Leioa, Spain; ^2^ Cell Therapy, Stem Cells and Tissues Group, Biocruces Bizkaia Health Research Institute, Barakaldo, Spain; ^3^ Research Unit, Basque Centre for Blood Transfusion and Human Tissues, Galdakao, Spain; ^4^ Red Española de Terapias Avanzadas (TERAV), Redes de Investigación Cooperativa Orientadas a Resultados en Salud (RICORS RD21/0017/0024), Instituto de Salud Carlos III (ISCIII), Madrid, Spain; ^5^ Red de Inmunoterapia del Cáncer “REINCA” (RED2022-134831-T), Madrid, Spain; ^6^ Basque Foundation for Science, Ikerbasque, Bilbao, Spain

**Keywords:** natural killer cells, NK, CAR-NK, surface potential, biomaterials, immunotherapy

## Abstract

Natural killer (NK) cells are lymphocytes of the innate immune system that play a key role in the elimination of tumor and virus-infected cells. Unlike T cells, NK cell activation is governed by their direct interaction with target cells via the inhibitory and activating receptors present on their cytoplasmic membrane. The simplicity of this activation mechanism has allowed the development of immunotherapies based on the transduction of NK cells with CAR (chimeric antigen receptor) constructs for the treatment of cancer. Despite the advantages of CAR-NK therapy over CAR-T, including their inability to cause graft-versus-host disease in allogenic therapies, a deeper understanding of the impact of their handling is needed in order to increase their functionality and applicability. With that in mind, the present work critically examines the steps required for NK cell isolation, expansion and storage, and analyze the response of the NK cells to these manipulations. The results show that magnetic-assisted cell sorting, traditionally used for NK isolation, increases the CD16+ population of NK cultures only if the protocol includes both, antibody incubation and passage through the isolation column. Furthermore, based on the importance of surface potential on cellular responses, the influence of surfaces with different net surface charge on NK cells has been evaluated, showing that NK cells displayed higher proliferation rates on charged surfaces than on non-charged ones. The present work highlights the relevance of NK cells manipulation for improving the applicability and effectiveness of NK cell-based therapies.

## 1 Introduction

The infusion of T cells engineered to express chimeric antigen receptors (CARs) was first authorized by the United States Food and Drug Administration (FDA) to treat B-cell acute lymphoblastic leukemia (ALL) in the year 2017 (1, 2). Although CAR-T technology has demonstrated success in treating several cancer types (3), it presents certain limitations such as occasionally causing cytokine release syndrome (CRS) or immune effector cell-associated neurotoxicity syndrome (ICANS) (4). The superior ability to kill malignant cells without causing the above drawbacks led to the modification of natural killer (NK) cells with CAR constructs as an alternative cell-based antitumoral therapy. NK cells are large granular cells of the immune system constituting approximately 10–15% of circulating lymphocytes in blood (5). These cells are part of the innate immune system, and consequently kill cancerous and virally infected cells with no prior priming requirement (6, 7).

The surface marker phenotype defining human NK cells within the lymphocyte population is characterized by a lack of CD3 (present in T cells) and expression of CD56, which is a 140-kDa isoform of the neural cell adhesion molecule (NCAM) (8). The inhibitory and activating receptors present in the membrane of NK cells and the integration of signals transmitted by them determines their response (9). Among these receptors, CD16 (FcγRIIIa) triggers the antibody dependent cytotoxicity (ADCC) response, which is a very powerful activating signal (10). In turn, CD57 expression is associated with more mature NK cells, and its expression increases with age and is associated with chronic infection (11). The simultaneous expression of CD57 and NKG2C, an activating receptor that forms heterodimers with CD94 that recognize HLA-E (12), has been associated with a memory-like NK cell population believed to confer resistance to certain viral infections (13).

Functional NK cells can be isolated from whole peripheral adult blood (AB) or umbilical cord blood (CB) by immunomagnetic negative selection (14, 15). However, this approach renders a limited number of cells that are difficult to expand. In order to overcome this limitation CD34+ hematopoietic progenitors from CB or derived from induced pluripotent stem cells (iPSCs) have as well been proposed as alternative sources of functional NK cells (16–20). In addition to primary NK cells, a number of studies use NK-92 cells, an established cell line that has also been approved by the FDA for its use in clinical trials (21). Expansion protocols for the production of large NK cell populations are based on the use of feeder cells and optimized culture media. The presence of naturally expressed or genetically engineered receptors on the membrane of feeder cells promotes the proliferation and activation of NK cells. The most frequently used feeder cells include irradiated PBMCs, Epstein-Barr lymphoblastoid cell line (EBV-LCL) and K562 lymphoblast cells (22, 23). Regarding soluble factors, the addition of the cytokines IL-2 and IL-15 has been repeatedly shown to increase the number of NK cells *in vitro*. This has inspired the genetic modification of K562 cells to express membrane-bound IL-15 (mIL-15) or IL-21 (mIL-21). The resulting combination of the activating signals naturally provided by the K562 cell line and the co-stimulation triggered by the cytokines has shown to promote NK cell proliferation and expansion of cytotoxic NK cells (22, 24–26). Nevertheless, the relevance of other factors, such as the physicochemical properties of the culture dishes used for NK cell expansion, remain largely unexplored. Electrostatic interactions, including salt-bridges and hydrogen-bonds, are forces that define the biomolecular interplay. Consequently, using materials that have a specific surface potential can significantly influence the reaction of biological materials that come into contact with them. In fact, in order to promote cell adhesion, standard polystyrene cell culture plastic is modified to become negative in aqueous solution (27). In turn, for cells that prefer positive surfaces, polystyrene is generally coated with synthetic polymers, such as poly-D-lysine. While the precise cause of such preference for electrically charged surfaces is unclear, the dielectric nature and ionic conducting properties of the cell membrane (28) would be responsible for the influence of environmental electric cues in cell types that do not typically spread and attach to surfaces, through cell-material contacts or via factor immobilization. For their use in clinical practice NK isolation and expansion protocols that follow GMP (Good Manufacturing Practice) conditions must be followed. These protocols include a number of criteria including the use of closed cell culture systems and certified culture media (29). A commercially available GMP-compliant NK purification system is the CliniMACS Plus device (Miltenyi Biotec), which carries out a two-step purification procedure consisting of CD3+ cell depletion through magnetic immunolabeling followed by CD56+ NK enrichment. A more complex equipment called CliniMACS Prodigy (Miltenyi Biotec), performs, in addition to NK isolation, their expansion. By using this equipment, several groups have optimized NK cell expansion protocols for clinical purposes (30–32).

In the present work we critically explore how the manipulation steps required for the isolation, expansion and storage of NK cells might affect their subsequent clinical use. For this purpose, we have tested the impact of magnetic-assisted cell sorting (MACS) and examined the influence of electrical charge of culture wells, culture media and cryostorage on NK cells. The results show that the MACS isolation method increases the CD16+ population of NK cultures only if the protocols includes both, antibody incubation and passage through the isolation column. When cultured in contact with surfaces with a net charge, NK cells displayed higher proliferation rates on charged surfaces than on non-charged ones, in a process associated with homotypic NK cluster formation. Taken together, our work highlights the importance of several parameters, including the optimization of the isolation, expansion and storage methods, for the improvement of NK cell-based therapies.

## 2 Materials and methods

### 2.1 Cell isolation

Peripheral adult blood (AB) and umbilical cord blood (CB) samples from adult healthy individuals were collected, through the Basque Biobank (http://www.biobancovasco.org), with approval from the Basque Committee of Ethics and Clinical Research (number PI2014138) following the criteria of the European Directive 2004/33/EC Annex III. All study subjects were provided written informed consent. Peripheral blood mononuclear cells (PBMCs) from AB (three donors, noted as donor 1 (age 18, male), donor 2 (age 61, male) and donor 3 (age 21, female) and from CB (n=3), were obtained by density gradient using Ficoll Paque Plus (GE Healthcare). Next, peripheral blood NK cells (AB-NK) and cord blood NK cells (CB-NK) were purified using the ‘NK Cell Isolation Kit’ (130-092-657, Miltenyi Biotec), following the protocol described previously in (33). Briefly, 1x10^7^ PBMCs were sequentially incubated with the supplied cocktail of biotin-conjugated antibodies not expressed by NK cells (‘NK Cell Biotin-Antibody Cocktail’) and with the magnetic microbeads conjugated with the secondary antibodies (‘NK Cell MicroBead Cocktail’). Next, the suspension of labelled cells was added to the isolation column and the flow-through, containing the NK-enriched cell population, was collected. In the experiments conducted to determine the impact of the isolation process on NK cell activation, different quantities of the biotin-conjugated antibodies were used.

### 2.2 Culture conditions

Non-poled and poled β-PVDF films (PolyK Technologies), allowing to obtain average zero surface charge (non-poled; average surface potential 0V) and average positive or negative surface charge (poled; average surface potential of 6V and -4V, respectively) (34), were cut into 14 mm diameter circles and sterilized by immersion in 70% ethanol, PBS and sterile water for 5 minutes each. AB-NK and CB-NK cells were plated at a density of 5x10^5^ and 2x10^6^ cells per milliliter, respectively, onto 24-well ultra low attachment plates (Merck) where the different PVDF films were previously placed. As a control we used glass coverslips. Two different culture media were used: “*Custom NK medium*” composed of RPMI 1640 (Gibco) containing 10% human AB serum (Innovative Research), 1% penicillin/streptomycin (Gibco), 1% GlutaMAX (Gibco), 500 U/ml IL-2 and 20 ng/ml IL-15 and “*NK medium*” composed of NK MACS medium (Miltenyi Biotec) with 1% NK MACS supplement, 5% human AB serum (Innovative Research), and 500 U/ml IL-2 (Miltenyi Biotec). In turn, NK92 cells (ATCC, CRL-2407) were cultured at 3x10^5^ cells per milliliter using Myelocult H5100 (STEMCELLS) containing 12,6% horse serum (Gibco) and 100 IU/ml human IL-2 (Miltenyi Biotec).

Images of the cultures were taken at days 1, 7, 14 and 21, using a Leica Microsystems DMi8 inverted microscope. These images were used to estimate the number of aggregates and their projected area using ImageJ. For cell counting cell clusters were mechanically disaggregated, and the cultures were subsequently discarded.

### 2.3 Flow cytometry and immunofluorescence

For flow cytometry analysis, cells were washed with PBS containing 10% FBS and incubated for 30 minutes at 4°C for labeling with anti-CD3-FITC (BD Biosciences, clone OKT3), anti-CD56-PE (Miltenyi Biotec, clone MEM-188), anti-CD57-APC (Biolegend, clone NK-1), anti-CD16-BV421 (BD Biosciences, clone 3G8), anti-NKG2C-BV510 (BD Biosciences, clone 134591) and 7AAD. For each sample, 100.000 events were recorded using a FACS Canto II cytometer (BD Bioscience). Cell populations were analyzed using FlowJo v.X.0.7 (TreeStar Inc.). Viability of thawed CB-NK cells was quantified using the LIVE/DEAD Fixable Near-IR Dead Cell Stain Kit (Invitrogen) following the manufacturer’s instructions.

### 2.4 NK cells cryopreservation, thawing and functionality

CB-NK cells were cryopreserved using a freezing medium consisting of 50% Plasmalyte (Baxter International Inc.), 40% human AB serum and 10% DMSO (Thermo Scientific) at 2x10^6^ cells per ml per vial. NK cells were thawed in a bath at 37°C, centrifuged at 362*g* for 5 minutes and resuspended in “NK medium”. The next day, cells’ viability was checked by flow cytometry. In order to test their functionality, thawed CB-NK cells were co-cultured with K562 target cells at a ratio of 1:1 in a 24-well plate for 4 h at 37°C. At the beginning of the assay, anti-CD107a-BV421 (BD Biosciences, clone H4A3) was added in order to detect the degranulation activity of the effector cells against the target cells. Golgi Stop (BD Biosciences) (monensin) was added following the manufacturer’s protocol. After the incubation, cells were collected, washed, and labeled with anti-CD3-PerCP/Cy5.5 (Biolegend, clone SK7), anti-CD56-APC (Miltenyi Biotec, clone REA196) and analyzed using flow cytometry. Degranulating NK cells, characterized by the expression of CD107a were determined in the CD56+/CD3− cell population.

### 2.5 Statistical analysis

Statistical differences were evaluated using GraphPad Prism 6 (GraphPad Software, Inc.) with paired Student’s t-test.

## 3 Results

Natural killer (NK) cells are powerful effector cells that can be induced to selectively target tumor cells using CAR constructs (14). These therapies have already shown great potential for the treatment of hematological cancers, and once some challenges are resolved, they will certainly be an effective approach for the treatment of solid malignancies. In the following, we critically analyze a number of factors that, although classically neglected, can potentially influence the antitumor activity of NK cells.

### 3.1 NK cell isolation

**Figure 1:**
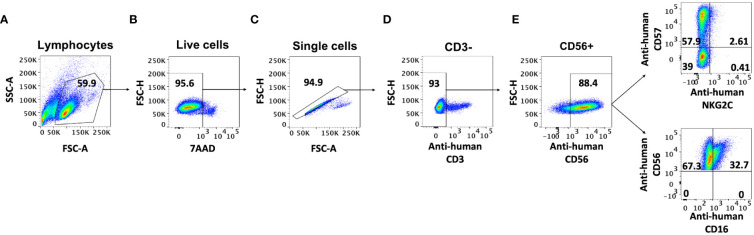
AB-NK cell labelling and gating strategy. Flow-cytometry gating strategy for AB-NK cells. **(A)** SSC-A and FSC-A were used to select the lymphocyte population; **(B)** FSC-H and 7AAD conjugated to PerCP-Cyanine5.5 were used to identify live cells; **(C)** FSC-H and FSC-A were used to select single cells; cell-surface markers CD3 **(D)** and CD56 **(E)** conjugated to the fluorescent dyes FITC and PE-Cy7 respectively, were used to sequentially identify NK cells; CD16 (cytotoxic NK), CD57 (mature NK), and NKG2C (active NK) conjugated to the fluorescent dyes Pacific Blue, Allophycocyanin (APC) and AmCyan respectively, were used to identify different NK cell populations.

**Figure 2:**
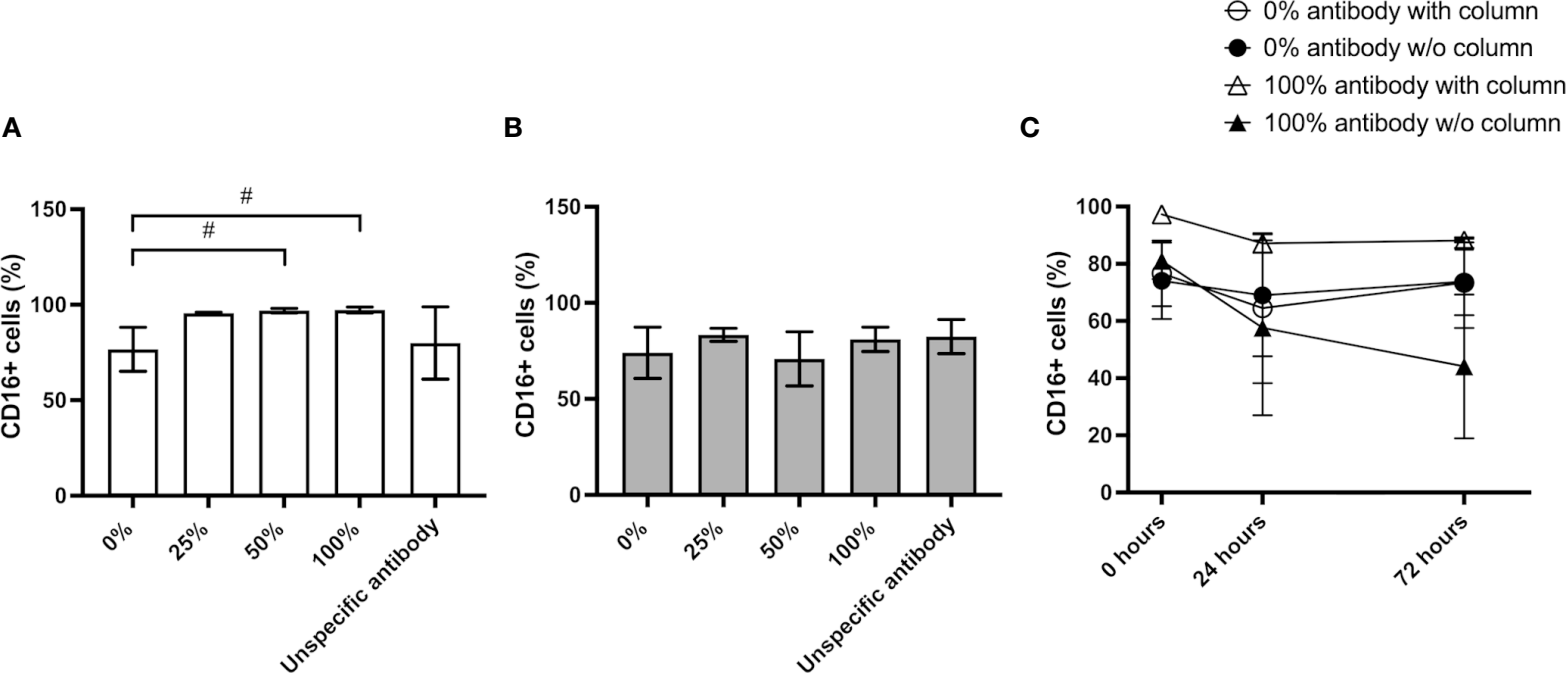
Impact of NK isolation on cell activation. **(A)** CD56+/CD16+ population resulting from the NK isolation protocol using isolation column and increasing amounts of antibody mix or an unspecific antibody. **(B)** CD56+/CD16+ population resulting from the incubation with antibodies without passaging the cells through the isolation column. **(C)** Comparison of the percentage of the CD56+/CD16+ NK population at different timepoints after the isolation. Results are expressed as mean ± SD (n=3). Statistical differences were analyzed by paired Student’s t-test. ^#^ indicates p-value<0.1.

The most widely used method to isolate NK cells from blood is the magnetic immunolabeling of non-NK cells for their depletion using a separation column placed in a magnetic field. Although this technique results in high NK enrichment rates, as revealed by CD56+/CD3- cytometry analysis (**Figure 1**), it has been suggested that the isolation process increases the expression of the surface markers CD57, NKG2C, and CD16 in the NK cells (13). To clarify how this cell activation occurs and determine its persistence over time, we have performed a series of experiments in which different steps of the isolation procedure were omitted and the effect on the cells was estimated using flow cytometry. Firstly, we performed the isolation procedure with different quantities of the biotin-conjugated antibodies not expressed by NK cells (‘NK Cell Biotin-Antibody Cocktail’) and analyzed the presence of CD16+ cells in the resulting population. The results show a consistent increase in the CD16+ population in all samples isolated with antibodies, with a slight increase at higher antibody concentrations (**Figure 2A**). Nevertheless, the addition of an antibody different to the supplied with the isolation kit did not result in an increased number of CD16+ cells (**Figure 2A**). We further tested whether NK cells could be activated just by their passage through the isolation column. No change in the resulting CD16+ population was observed. The results reveal that without isolation column, the antibody-dependent activation does not occur, and the CD16+ population remains similar before and after the manipulation (**Figure 2B**). The activated state obtained with the full isolation protocol persists over the next few days, remaining the CD16+ population in the cultures that were subjected to the complete isolation protocol (100% antibody mix and column) larger than in the other conditions for at least 3 days (**Figure 2C**).

The established NK cell line NK-92 is used as an alternative to primary NK cells in both research and cancer immunotherapy (35). Although for their use NK-92 cells are not necessarily isolated using immunolabelling, we further investigated the effects of the isolation procedure on these cells (**Supplementary Figure 1**). Unlike AB-NK cells, NK-92 are insensitive to all conditions tested and the CD16+ population remains very low regardless of the procedure (**Supplementary Figure 2**).

### 3.2 Cell expansion

**Figure 3:**
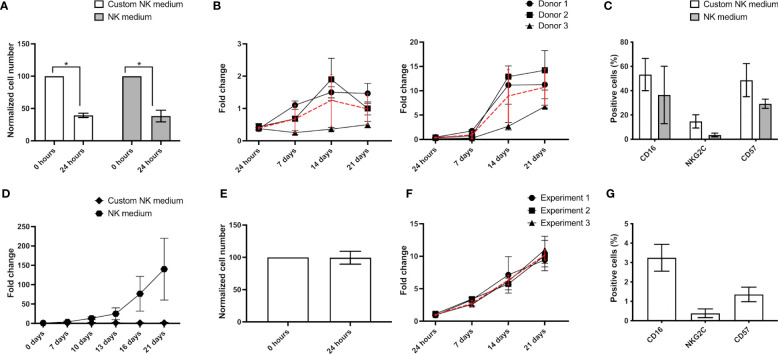
NK cell expansion. **(A)** After 24 hours culture AB-NK cell number is strongly reduced using “Custom NK medium” (white bars) or “NK medium” (grey bars). **(B)** Growth curves and average growth (red curves) of AB-NK cultured using “Custom NK medium” (left panel) and “NK medium” (right panel). **(C)** Flow cytometry analysis reveals no differences in the expression of surface markers in AB-NK cells expanded using either media. **(D)** Growth curves of CB-NK cells cultured using “Custom NK medium” and “NK medium”. **(E)** A majority of NK-92 cells remain alive 24 hours after seeding. **(F)** Growth curves of 3 independent experiment of NK-92 and average growth (red curves). **(G)** Marker expression after NK-92 expansion analyzed by flow cytometry. All of results are expressed as mean ± SD (n=3). Statistical differences were analyzed by paired Student’s t-test. * indicates p-value<0.05.

For NK cell expansion we have tested two different culture media, one based on RPMI 1640 with 500 U/ml IL-2 and 20 ng/ml IL-15, a medium classically used to culture human lymphocytes (hereby called “*Custom NK medium*”), and a media commercialized by Miltenyi Biotec (NK MACS medium with 500 U/ml IL-2, hereby called “*NK medium*”). Independently of the media used, over 50% of the initially plated AB-NK cells were lost during the first 24 hours (**Figure 3A**). Nevertheless, over the next 7 days the number of NK cells increased in both media, displaying a significantly faster proliferation rate in “NK medium” (**Figure 3B**). Specifically, in this medium, cell number approximately increased by 10-fold after 21 days, while in “Custom NK medium” the number of NK cells just doubled in the same period (**Figure 3B**). The expression of surface markers in NK cells expanded using either media did not display significant differences after 21 days in culture (**Figure 3C**). CB-NK cells cultured with “Custom NK medium” and “NK medium” exhibited similar trends, being however their proliferation significantly faster. Specifically, cultures with “Custom NK medium” reached nearly 5x10^6^ NK cells at day 21, while cultures with “NK medium” reached cell counts up to 60x10^6^ cells in the worst case and 360x10^6^ cells in the best case. In terms of fold change, this indicates an average increase of 120-fold at day 21 of culture for CB-NK cells cultured with “NK medium” (**Figure 3D**). In turn, compared with AB-NK cells, the number of NK-92 cells did not decrease 24 hours after plating (**Figure 3E**) and their growth curves displayed minimal variations between experiments (black lines in **Figure 3F**). Surface marker analysis reveals a residual population of CD16+ and CD57+ cells in the cultures with NK-92 after 21 days in culture (**Figure 3G**).

**Figure 4:**
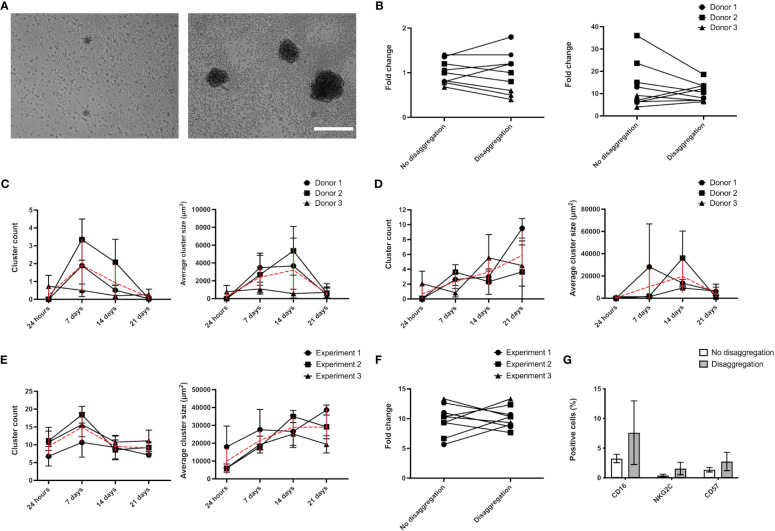
Analysis of NK cluster formation. **(A)** Representative images of AB-NK clusters in “Custom NK medium” (left image) and “NK medium” (right image) after 14 days culture. **(B)** AB-NK cell count after 21 days culture with and without weekly disaggregation of clusters using “Custom NK medium” (left panel) or “NK medium” (right panel). Average AB-NK cluster number (left panels) and size (right panels) over time using “Custom NK medium” **(C)** or “NK medium” **(D)**. **(E)** Average number and size of clusters formed in NK-92 cultures over time. **(F)** Impact of cluster disaggregation in NK-92 cultures. **(G)** Analysis of surface marker expression of NK-92 cultures with and without disaggregation. Results are expressed as mean ± SD (n=3). In **(C–E)** red line represents the average value. Scale bar in A represents 400 μm.

It has been proposed that homotypic cell clustering favors NK proliferation via a mechanism called trans-presentation (36). To test whether the increased proliferation displayed by AB-NK cells in “NK medium” is related with this process, we quantified the impact of a weekly mechanical disaggregation of the clusters that form in the cultures (**Figure 4A**). Initially, we examined the variation in the proliferation rate between disaggregated and intact cultures. However, after conducting a paired Student’s t-test, we found no statistically significant differences (**Figure 4B**). Next, we analyzed the dynamics of NK cell clustering by quantifying the number of clusters and the projected area covered by them over the culture time. Using “Custom NK medium”, we observed an increase in the number and average size of the clusters during the first weeks of culture and a decrease at day 21 (**Figure 4C**). Similarly, in “NK medium” AB-NK cells displayed a peak in cluster size after approximately 2 weeks culture (**Figure 4D**). However, in this case the cluster count did not decline during the three-week period that was analyzed (**Figure 4D**). Regarding NK-92 cells, clusters grow larger over time, but their number decreases after 7 days of culture, perhaps due to the aggregation of smaller clusters into larger ones (**Figure 4E**). Regarding NK-92 cluster disaggregation did not show statistically significant changes on fold expansion or in the expression of surface markers (**Figures 4F, G**).

### 3.3 Surface charge

**Figure 5:**
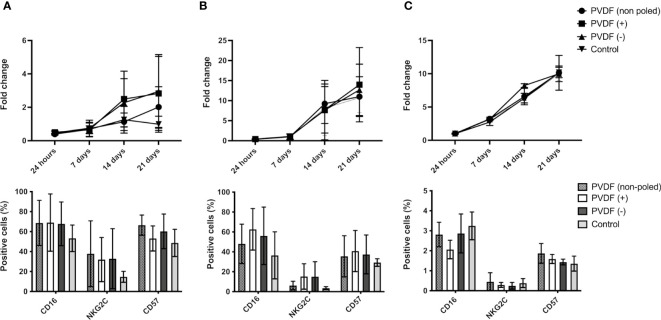
Effect of surface potential on NK proliferation and phenotype. Growth of AB-NK cells with “Custom NK medium” **(A)** and “NK medium” **(B)** in contact with PVDF of different polarities (top panels) and surface marker analysis after cell expansion (bottom panels). **(C)** Growth of NK-92 in contact with PVDF of different polarities (top panel) and surface marker analysis after cell expansion (bottom panel). Results are expressed as mean ± SD (n=3).

The impact of the physicochemical properties of the microenvironment on lymphocytes remains largely undescribed. However, properties such as the surface potential of the materials used in cell culture are determinant of cell response (37). It is hypothesized that surface charge may also affect non-adhering cells due to the long-range electrostatic interactions, via factor immobilization that might impact their biological activity or via short-term cell-substrate contacts. To study the effect of the surface potential of the culture containers on AB-NKs, cells were seeded on polarized and non-polarized PVDF discs with net positive (6V), negative (-4V) or neutral (0V) surface charge (34), and cultured using “Custom NK medium” or “NK medium” for 21 days. Results show an increase in AB-NK cell proliferation in those cultures using “Custom NK medium” that included a PVDF surface with a net charge, either positive or negative, compared to the corresponding control cultures (**Figure 5A**, top panel). Nevertheless, these changes were not as evident when cells were cultured using “NK medium” (**Figure 5B**, top panel) and were not observed in NK-92 cell cultures (**Figure 5C**, top panel).

We further analyzed the expression of surface markers related with cytotoxicity (CD16), maturation (CD57) and activation (NKG2C) in CD56+ NK cells after 21 days culture in contact with PVDF surfaces with different surface potentials. AB-NK cultures using “Custom NK medium”, and NK-92 cultures in contact with electrically charged or neutral PVDF displayed no significant changes in the expression of the above-mentioned markers (**Figures 5A, C**, bottom panels). In turn, using “NK medium”, the NKG2C+ population was slightly larger in cultures in contact with poled PVDF. A similar trend was observed for the CD57+ and CD16+ populations (**Figure 5B**, bottom panel).

**Figure 6:**
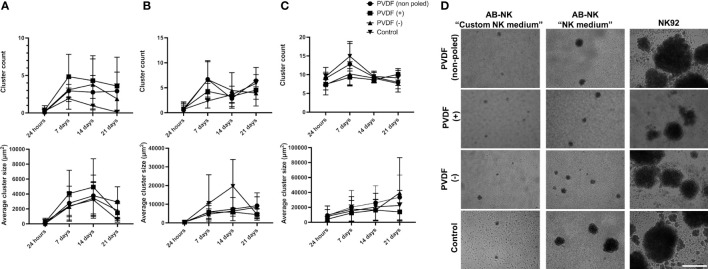
NK clustering in presence of different surface charges. Cluster number and average size of AB-NK cells cultured on surfaces with a net positive, negative or neutral charge using “Custom NK medium” **(A)** or “NK medium” **(B)**. **(C)** Cluster formation and average size of the clusters in NK-92 cultures. Results are expressed as mean ± SD (n=3). **(D)** Representative images of AB-NK and NK-92 clusters after 14 days culture. Scale bar represents 400 µm.

We further analyzed the dynamics of NK cluster formation in the presence of polarized PVDF surfaces. In case of “Custom NK medium”, the higher proliferation rate of AB-NKs on positive and negative surfaces was accompanied by an increased number of clusters (**Figures 6A, D**), which, as it was the case of the control conditions, displayed a peak size between 7 and 14 days of culture. In turn, these cells cultured with “NK medium” and NK-92 cultures displayed no differences between the different conditions (**Figures 6B, C** and **Supplementary Figures 3-6**).

**Figure 7:**
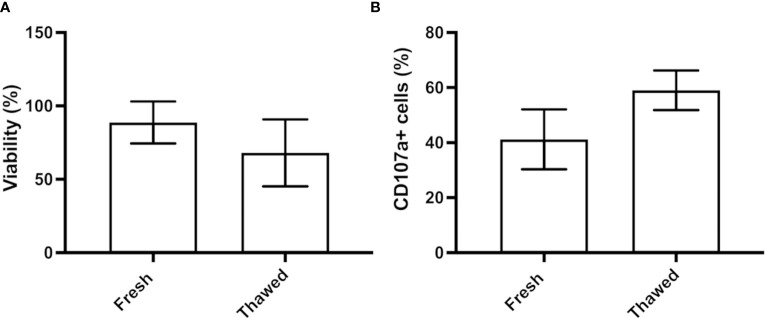
NK cell cryopreservation. **(A)** Percentage of viability of CB-NK cells before and after their cryopreservation. **(B)** Flow cytometry analysis of the functionality of fresh and thawed CB-NK cells measured by degranulation assay against K562 target cells. Results expressed as mean ± SD (n=3).

For their use as an off-the-shelf product, after their expansion NK cells should be cryopreserved. We therefore analyzed the impact of freezing using a medium composed of Plasmalyte, human serum and DMSO (13) on CB-NK cell survival and functionality. Upon thawing, CB-NK cells exhibited a viability of 68%, compared to the 88.7% viability found in the fresh culture (**Figure 7A**). Next, we incubated the cells before and after the cryostorage with K562 target cells (ratio 1:1) and analyzed the expression of CD107a, a marker of degranulation. Although no statistical differences were found, the expression was slightly higher in thawed cells than in fresh ones (**Figure 7B**).

## 4 Discussion

Natural killer (NK) cells represent a promising approach for allogenic CAR immunotherapy thanks to their ability to kill cancer cells and their low tendency to provoke graft-versus-host disease. Nevertheless, their low count in blood, representing around 10% of all lymphocytes, and limited expandability are significant challenges for developing off-the-shelf CAR-NK therapies. In this context, progress towards reducing batch-to-batch variability and enhancing number and functionality of isolated NK cells is essential. With this in mind, in the current work, we have conducted a thorough analysis of the effects of all necessary manipulation steps required for the clinical application of NK cells, namely isolation, expansion and storage, and examined the mechanisms underlying the observed cellular response.

In general terms, large differences are found in the results obtained using AB-NK cells from different donors, which, together with the replicability of the experiments with cells from the same source (same donor or NK-92 cells), indicates donor-to-donor variability as a likely cause. Several studies have investigated the impact of donor characteristics on NK cell function. For instance, it has been observed that the overall NK cell count grows with age. Indeed, the percentage of CD56^bright^ NK cells, that is the subpopulation of NK cells that displays large amounts of CD56 marker and produce high levels of inflammatory cytokines, increases. In turn, the CD56^dim^ subpopulation, which displays low amounts of CD56 and contain abundant lytic granules, decreases (38–40), ascribed to an age-related decline in IL-2 expression (41). It has been also suggested that NKs from older donors display lower cytotoxicity (42). Another potential source of variability is the sex of the donor. Although the mechanisms are not completely understood, it has been shown that in males the antiviral activity of these cells is impaired (43).

NK cells express a number of activating, co-stimulatory and inhibitory receptors that regulate their activity. One of these receptors is CD16 (also known as Fc-gamma receptor IIIa or FcγRIIIa), which binds to the Fc-region of antibodies and promotes antibody-dependent cell-mediated cytotoxicity (ADCC). ADCC has been identified in a number of cells of the innate immune system, but is of particular importance in NK cells because they do not express the inhibitory FcγRIIb isoform (44, 45). Our results show that the antibodies used during the isolation of NK cells from blood, cause the increase of the population of CD16+ cells, probably through a mechanism that requires a high density of antibodies in a specific arrangement in order to take place (46). This may explain the inability of non-specific and therefore unbound antibodies and the need for passage through the column for ADCC NK cell activation. In turn, only a small proportion of NK-92 cells express CD16 (47) and consequently these cells are not activated via antibodies. Other alternatives for NK isolation that do not require antibodies, such as microfluidic systems, which have already proven to be efficient in high-resolution separation and sorting of blood cells down to the single-cell level (48), could be used to avoid this activation for those applications that require uncommitted cells.

The resting potential, which refers to the electrical potential difference across the plasma membrane, is recognized as one of the physico-chemical properties of cancer cells that can affect the function of NK cells (49). Our experiments did not discover significant changes in the surface marker fingerprint of NK cells cultured on surfaces with distinct net electric charges/potential, however, we observed an increased proliferation rate in NK cells cultured with “Custom NK medium” and in contact with PVDF surfaces with a net electric charge. Multicellular cluster formation is known to potentiate IL-2-driven activation of NK cells, and consequently promote their survival and proliferation (47). Our experiments confirm the correlation between increased cluster formation and higher cell expansion, and further establish a link of the process with the surface potential of the physical microenvironment where the cells reside (49). In fact, *in vivo*, NK clustering occurs in the lymph nodes (50), an environment characterized by the accumulation of negatively charged glycosaminoglycans (51). The observed dependency of the *in vitro* clustering process on the culture medium used, suggests that further molecular mechanisms might be involved. Additionally, disaggregation experiments show that careful cell handling and the promotion of homotypic cell clustering by engineering the culture microenvironment, represents an appropriate strategy to support NK cell expansion. However, it should be noted that having more proliferative cells, and thus a greater number of cells, is beneficial, as long as their functionality is not compromised by the expansion. However, it is known that not all NK cell subtypes are equally effective against cancer cells. For example, studies have indicated that the NK cell subsets most successful in fighting acute lymphoblastic leukaemia (ALL) expressed NKG2A, while NK cells expressing CD57 and/or KIR demonstrated greater efficacy against acute myeloid leukaemia (AML) (52).

For their use as an off-the-shelf product, after their expansion NK cells should be stored until their infusion. Here, we tested the impact of cryopreservation of NK cell functionality. Interestingly, the degranulation of previously cryopreserved NK cells was higher than that of fresh NK cells. Previous investigations have shown a good recovery of NK cells activity after freezing (53), being however their ability to kill tumor cells of solid malignancies impaired by their reduced migration capacity (54). Although the reason for the improved degranulation of thawed CB-NKs is not clear, it is possible that the process of freeze-thawing has selected the most active NK cells.

## 5 Conclusions and outlook

In recent years the development of new off-the-shelf NK-based approaches for cancer treatment has gained momentum. Nevertheless, the production of such cell treatments still encounters a number of limitations that need to be addressed before their widespread use in clinical practice. In the present work, we have critically examined a number of elements of the initial steps of NK handling that are required for their application. While the statistical analysis of some of these factors is impaired by the large inter-donor variability, our results point to an effect of the isolation method, culture media, cell clustering and surface charge on NK cell activity. Specifically, we observed that immunomagnetic NK separation leads to activation of NK cells and, although the complete elimination of antibodies from the isolation procedure might not be possible, it is important to note that such activation occurs and could affect downstream NK functions. With respect to surface charge, further experiments should decipher the mechanism by which surface potential enhances expansion of NK cells in “Custom NK medium” to exploit this and further enhance the already superior expansion obtained with “NK medium”. This may also help to understand the molecular mechanism by which NK cell clustering occurs, and thereby promote it and increase the rate of NK expansion.

## Data availability statement

The raw data supporting the conclusions of this article will be made available by the authors, without undue reservation.

## Ethics statement

Blood samples were collected through the Basque Biobank (http://www.biobancovasco.org) in accordance with a protocol approved by the Basque Committee of Ethics and Clinical Research (number PI2014138).

## Author contributions

SM-I and LH carried out the experiments. SS, and MV and CE participated in the planning of the experiments and in the analysis of the results. SL-M and US conceived the experiments, analyzed the results and supervised the work. All authors contributed to the final version of the manuscript.
