# Supplementary material for: Analysis of the impact of handling and culture on the expansion and functionality of NK cells
Source: Front Immunol. 2023 Aug 11;14:1225549. doi: 10.3389/fimmu.2023.1225549 (PMC10451065; doi:10.3389/fimmu.2023.1225549)
Supplement: Supplementary file 1 [file DataSheet_1.docx]

Supplementary Material

Analysis of the impact of handling and culture on the expansion and functionality of NK cells

Sara Martin-Iglesias^1^, Lara Herrera^2,3^, Silvia Santos^2,3^ , Miguel Ángel Vesga^2,3^ , Cristina Eguizabal^2,3^, Senentxu Lanceros-Mendez^1,5^, Unai Silvan^1,5*^

* Correspondence:

Unai Silvan
[unai.silvan@bcmaterials.net](mailto:unai.silvan@bcmaterials.net)

**Supplementary Figures:**

**
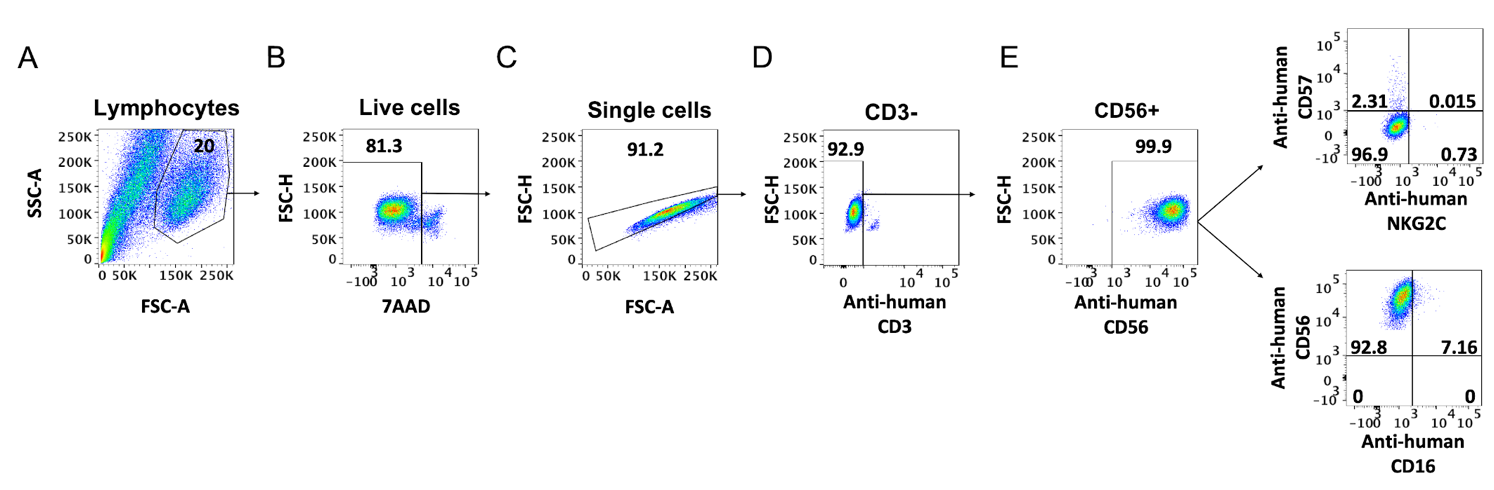
**

**Supplementary figure 1. NK-92 gating strategy.** Flow-cytometry gating strategy for NK-92. (A) SSC-A and FSC-A were used to select the lymphocyte population; (B) FSC-H and 7AAD conjugated to PerCP-Cyanine5.5, identify live cells; (C) FSC-H and FSC-A, show singlets; cell-surface markers CD3 (D) and CD56 (E) conjugated to the fluorescent dyes FITC and PE-Cy7 respectively, were used to sequentially identify NK cells; CD16 (cytotoxic NK), CD57 (mature NK), and NKG2C (active NK) conjugated to the fluorescent dyes Pacific Blue, Allophycocyanin (APC) and AmCyan respectively, were used to identify different NK cells populations.


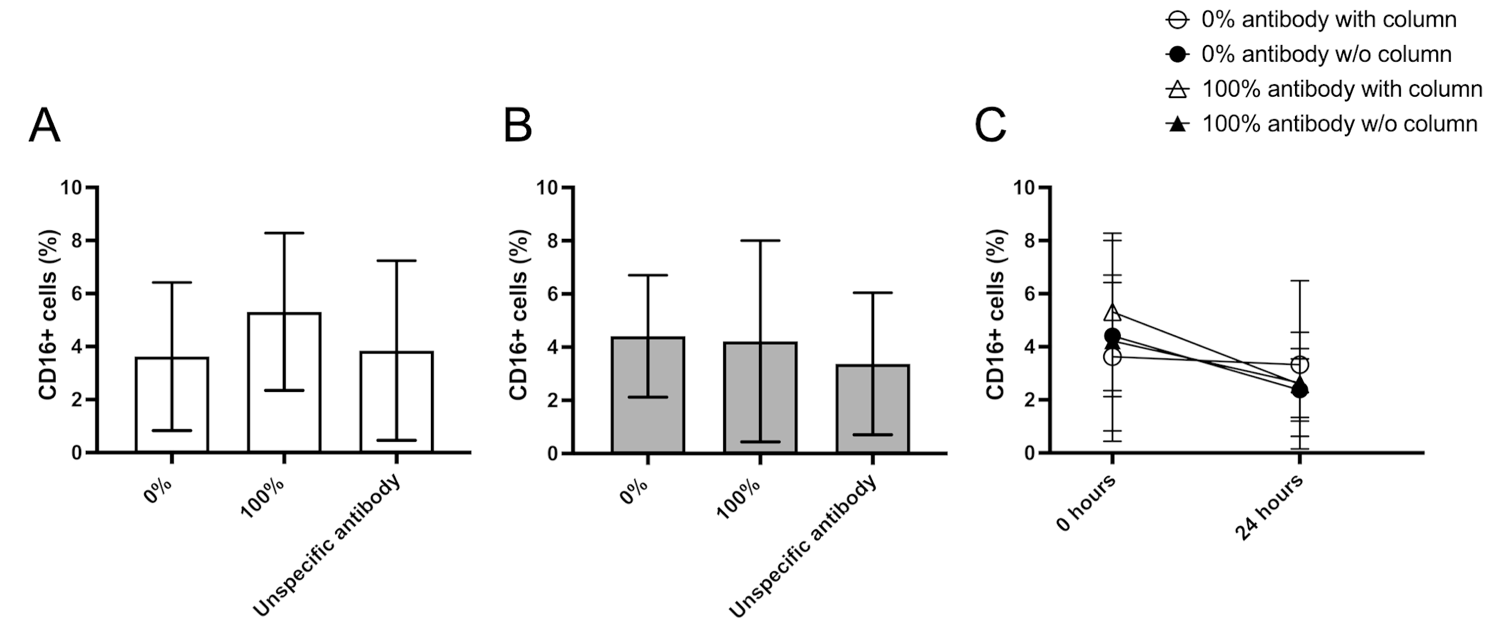


**Supplementary figure 2.** **NK-92 cell response to the NK isolation protocol**. CD56/CD16+ population resulting from the isolation protocol with (A) and without isolation column (B). (C) Comparison of the percentage of CD16+ cells directly after the isolation and 24 later. Results expressed as mean ± SD (n=3).


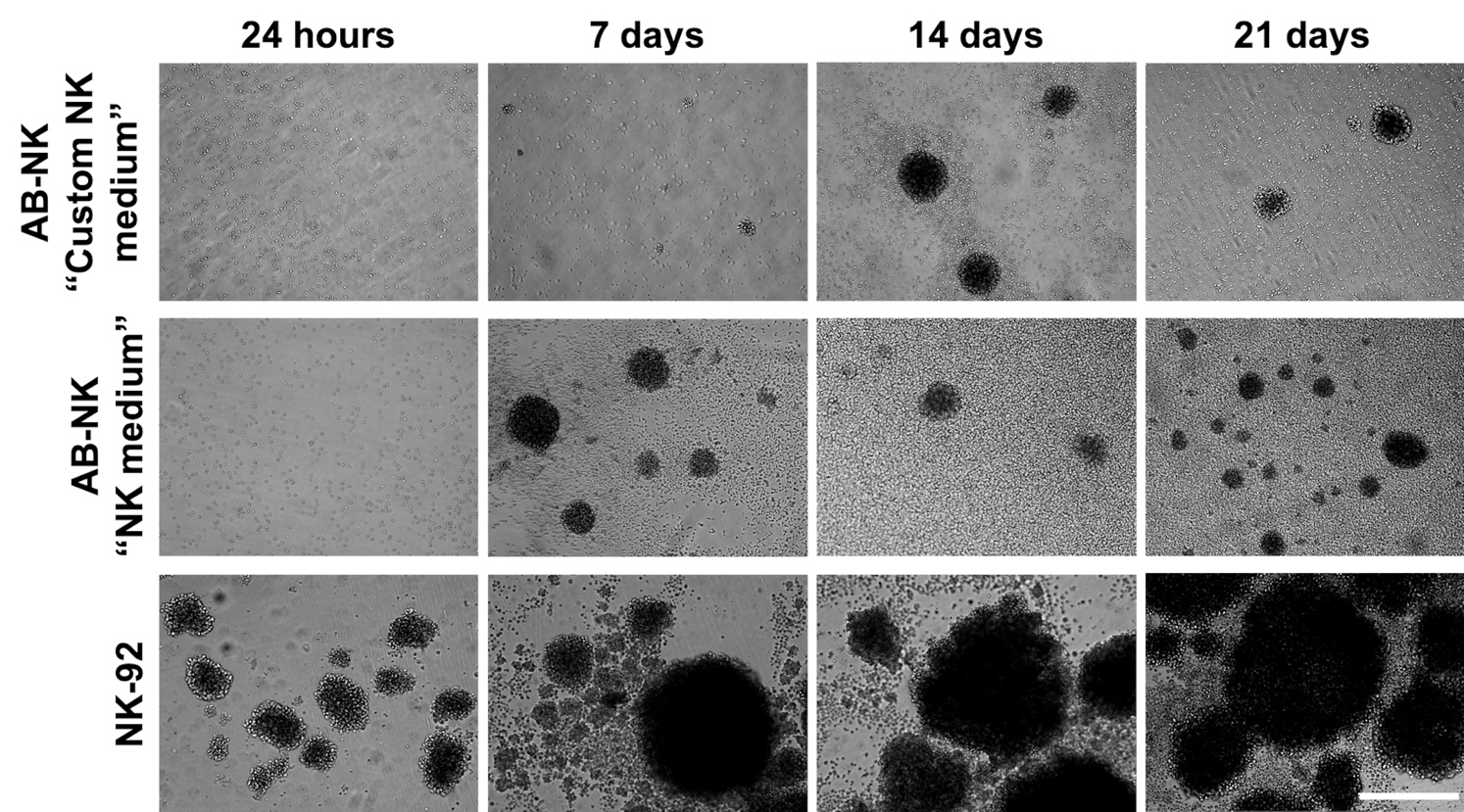


**Supplementary figure 3. NK cluster formation over time on PVDF with no net charge.** AB-NK cultured with “Custom NK medium” (upper row) or “NK medium” (middle row), and NK-92 with “Myelocult medium” (bottom row). Scale bar represents 400 µm.


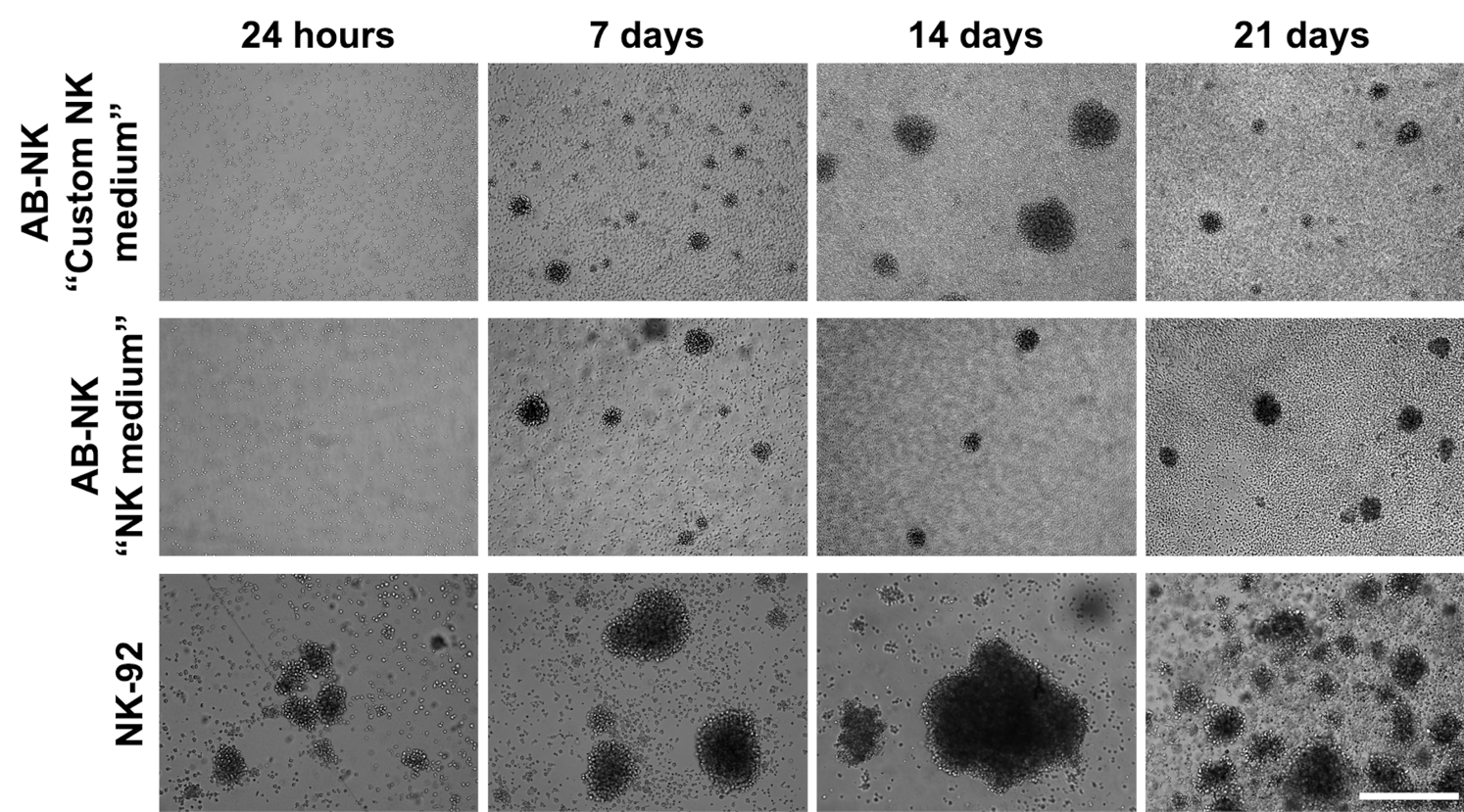


**Supplementary figure 4. NK cluster formation over time on PVDF with net positive charge**. AB-NK cultured with “Custom NK medium” (upper row) or “NK medium” (middle row), and NK-92 with “Myelocult medium” (bottom row). Scale bar represents 400 µm.


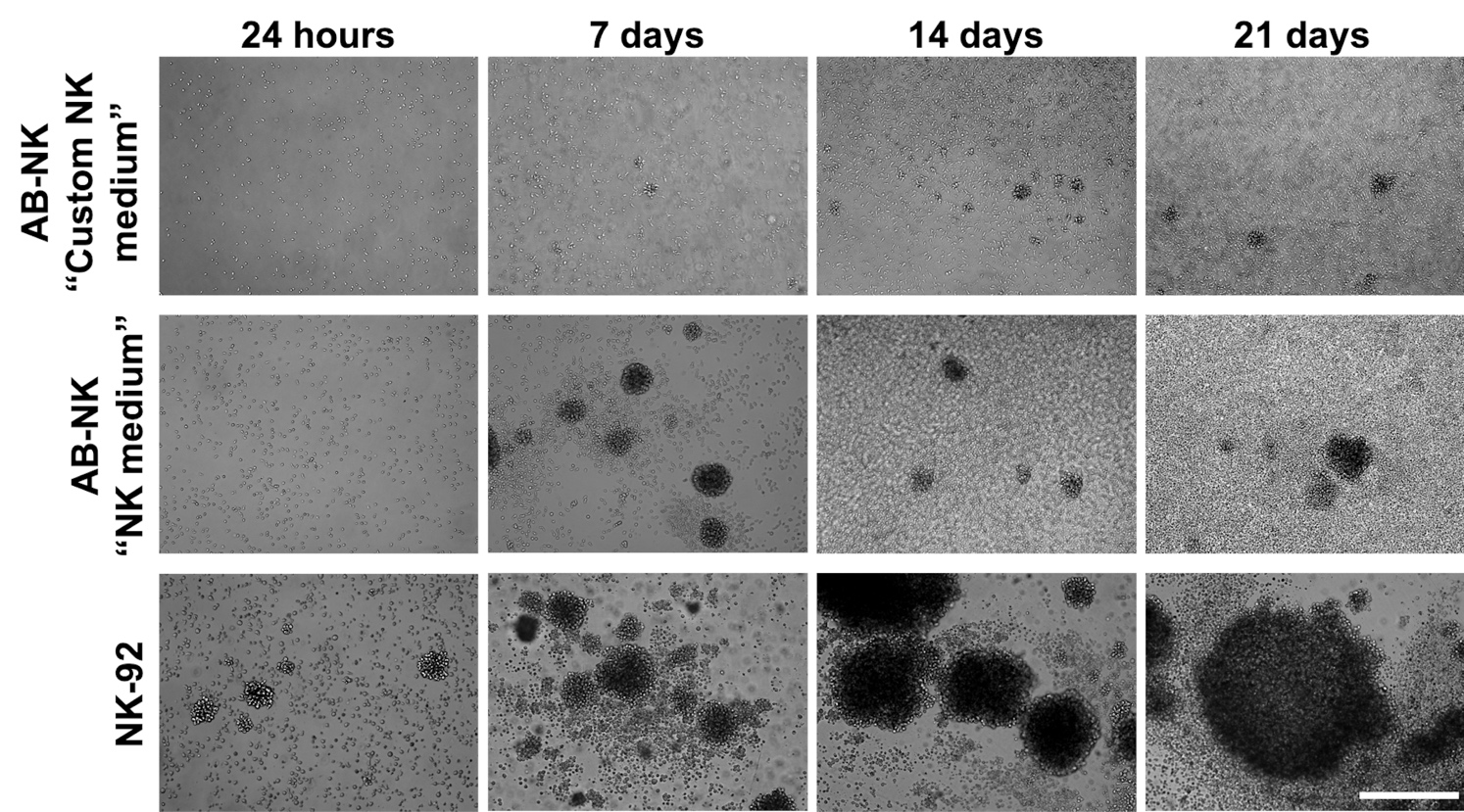


**Supplementary figure 5. NK cluster formation over time on PVDF with negative net charge**. AB-NK cultured with “Custom NK medium” (upper row) or “NK medium” (middle row), and NK-92 with “Myelocult medium” (bottom row). Scale bar represents 400 µm.


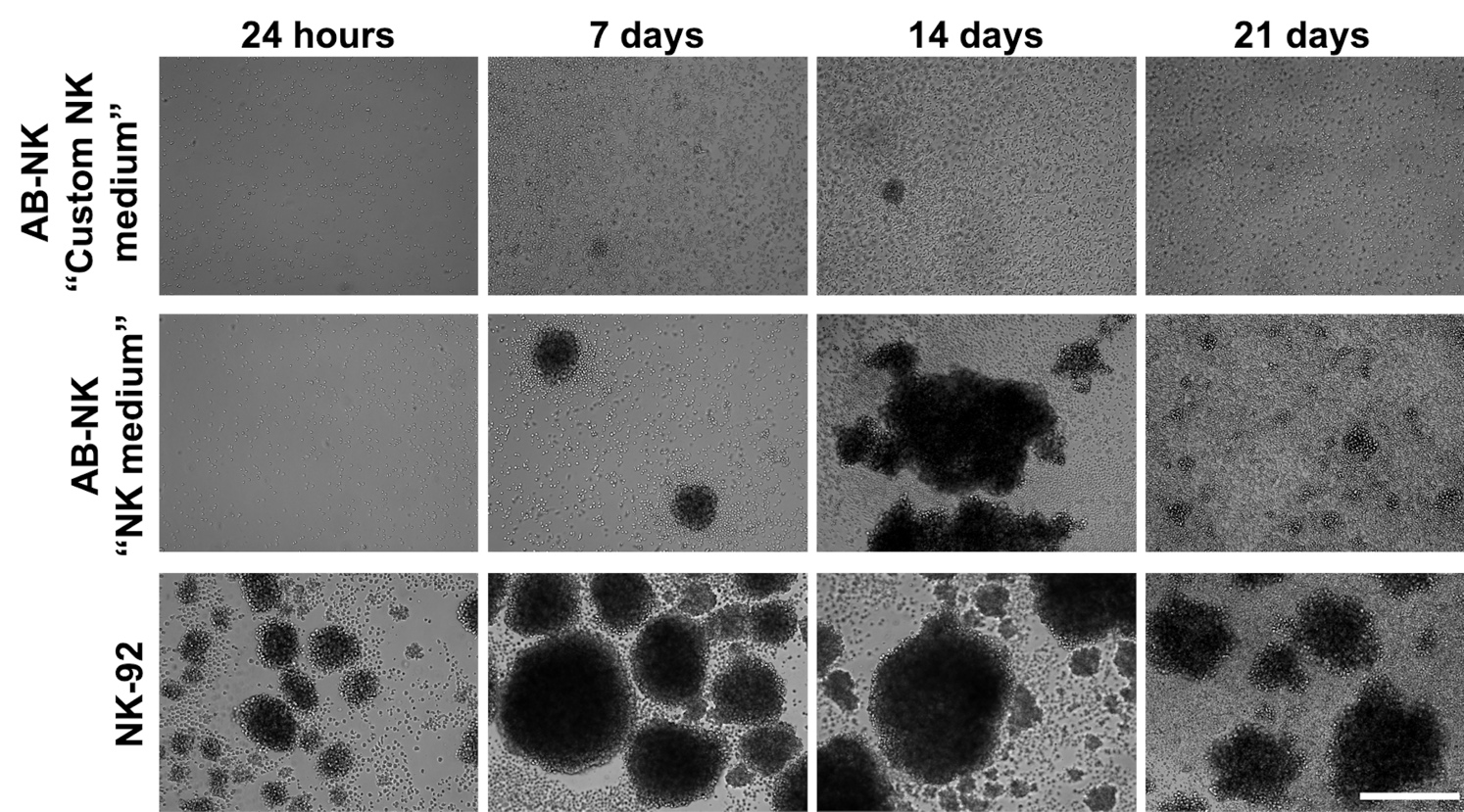


**Supplementary figure 6. NK cluster formation over time on control culture conditions.** AB-NK cultured with “Custom NK medium” (upper row) or “NK medium” (middle row), and NK-92 with “Myelocult medium” (bottom row). Scale bar represents 400 µm.
